# Supplementary material for: Diversity of soil bacterial communities in response to fonio (Digitaria exilis Stapf) genotypes and pedoclimatic conditions in Benin
Source: PLoS One. 2026 Jan 30;21(1):e0330794. doi: 10.1371/journal.pone.0330794 (PMC12857997; doi:10.1371/journal.pone.0330794)
Supplement: S1 Table — (DOCX) [file pone.0330794.s001.docx]

**S1 Table. Pedoclimatic condition effect on functions detected in the fonio rhizosphere bacterial community at KEGG level 3 pathways.**

| **KEGG level 3 functional categories** | **P value** | **Significance level** |
| --- | --- | --- |
| **Cellular processes** | 0.03 | * |
| **Environmental information processing** | 0.79 | NS |
| **Genetic information processing** | 0.009 | ** |
| **Human diseases** | 0.65 | NS |
| **Metabolism** | 0.06 | NS |
| **Organismal systems** | < 0.001 | *** |

**, ** and *** indicate the significance levels at p < 0.05 and 0.01 respectively, NS non-significant at p > 0.05.*
