# Supplementary material for: Diversity of soil bacterial communities in response to fonio (Digitaria exilis Stapf) genotypes and pedoclimatic conditions in Benin
Source: PLoS One. 2026 Jan 30;21(1):e0330794. doi: 10.1371/journal.pone.0330794 (PMC12857997; doi:10.1371/journal.pone.0330794)
Supplement: S2 Table — (DOCX) [file pone.0330794.s002.docx]

**S2 Table. Pedoclimatic condition effect on functions detected in the fonio rhizosphere bacterial community at KEGG level 2 pathways.**

| **KEGG level 2 functional categories** | **P value** | **Significance level** |
| --- | --- | --- |
| **Others** | 0.10 | NS |
| **Glycan biosynthesis and metabolism** | 0.01 | * |
| **Metabolism of other amino acids** | 0.06 | NS |
| **Cell growth and death** | 0.005 | ** |
| **Folding, sorting and degradation** | 0.007 | ** |
| **Metabolism of terpenoids and polyketides** | < 0.001 | *** |
| **Drug resistance antimicrobial** | 0.57 | NS |
| **Nucleotide metabolism** | 0.30 | NS |
| **Cell motility** | 0.14 | NS |
| **Xenobiotics biodegradation and metabolism** | 0.03 | * |
| **Replication and repair** | 0.001 | ** |
| **Lipid metabolism** | 0.98 | NS |
| **Energy metabolism** | 0.002 | ** |
| **Translation** | 0.05 | NS |
| **Metabolism of cofactors and vitamins** | 0.53 | NS |
| **Amino acid metabolism** | 0.19 | NS |
| **Carbohydrate metabolism** | < 0.001 | *** |
| **Cellular community of prokaryotes** | 0.04 | * |
| **Signal transduction** | 0.15 | NS |
| **Membrane transport** | 0.78 | NS |
| **Global and overview maps** | 0.22 | NS |

**, ** and *** indicate the significance levels at p < 0.05 and 0.01 respectively, NS non-significant at p > 0.05.* “Others” grouped level 2 KEGG pathways with a relative abundance of less than 1%.
