# Supplementary material for: Diversity of soil bacterial communities in response to fonio (Digitaria exilis Stapf) genotypes and pedoclimatic conditions in Benin
Source: PLoS One. 2026 Jan 30;21(1):e0330794. doi: 10.1371/journal.pone.0330794 (PMC12857997; doi:10.1371/journal.pone.0330794)
Supplement: Attachment — Submitted filename: Response to reviewers.docx [file pone.0330794.s003.docx]

**Manuscript : Diversity of soil bacterial communities in response to fonio (*Digitaria exilis* Stapf) genotypes and pedoclimatic conditions in Benin**

**Response to Editor' comments**

We thank the editor for his valuable and helpful comments on the manuscript. We provided below a point-by-point response to the comments.

**Editor’s comments**

**Comment #1**. Please ensure that your manuscript meets PLOS ONE's style requirements, including those for file naming. The PLOS ONE style templates can be found at

<https://journals.plos.org/plosone/s/file?id=wjVg/PLOSOne_formatting_sample_main_body.pdf> and <https://journals.plos.org/plosone/s/file?id=ba62/PLOSOne_formatting_sample_title_authors_affiliations.pdf>

**Answer #1**. The journal, PLOS ONE’s style requirements were checked, and the revised manuscript was corrected accordingly.

**Comment #**2. In your Methods section, please provide additional information regarding the permits you obtained for the work. Please ensure you have included the full name of the authority that approved the field site access and, if no permits were required, a brief statement explaining why.

**Answer #2.** Thank you for pointing this out. In the revised manuscript, we have specified in the Methods section (subheading: Study area and rhizosphere soil sampling method) the authority that approved access to the field sites, including the full names of the centers. We have also acknowledged them in the Acknowledgements section.

**The text now reads (lines 111-119): ‘’**

**National and local approval**

Before embarking on the fieldwork for sampling, the first author secured the approval of the academic committee of the École Doctorale des Sciences Agronomiques et de l'Eau (EDSAE) of the Faculty of Agricultural Sciences (FSA) of the University of Abomey-Calavi (UAC). The ethics committee that waived the study was the first author’s thesis committee at the University of Abomey-Calavi comprising professors Enoch G. Achigan-Dako, Adam Ahanchede, Guillaume Amadji. Prior to rhizosphere soil sampling, the objectives of the research project were presented to the local authorities of each village under investigation. The informed verbal consent of all local authorities was obtained before sample collection.’’

**Subheading: Study area and rhizosphere soil sampling method:**

**(lines 135 – 138)**

“These experimentations were carried out from June to August 2023 on fonio farmers’ fields and at the National Institute for Agricultural Research of Benin (INRAB) research field; permissions were obtained before installation and data collection.”

**Acknowledgements:**

**(Lines 513 – 516)**

“We thank the “Institut National des Recherches Agricoles du Bénin (INRAB)” and fonio farmers through “La Maison du Fonio” for giving their approval to implement our experimentation and collect the soil samples.”

**Comment #**3. Thank you for stating the following financial disclosure:
“This research was funded by French National Research Agency through the PEA-BIOVALOR (ANR-21-PEA2-0006) Biovalor project, within the framework of Tania L. I. Akponikpè’s PhD. “

Please state what role the funders took in the study. If the funders had no role, please state: "The funders had no role in study design, data collection and analysis, decision to publish, or preparation of the manuscript."
If this statement is not correct you must amend it as needed.
Please include this amended Role of Funder statement in your cover letter; we will change the online submission form on your behalf.

**Answer #3.** This statement was added to the cover letter revised as recommended:

“Tania L. I. Akponikpè’s PhD is a scholar of the University of Abomey-Calavi (Republic of Benin) and the Université de Lorraine (France). Her research work was funded by French National Research Agency through the PEA-BIOVALOR (ANR-21-PEA2-0006) Biovalor project. The funders had no role in study design, data collection and analysis, decision to publish, or preparation of the manuscript.”

**Comment #4.** When completing the data availability statement of the submission form, you indicated that you will make your data available on acceptance. We strongly recommend all authors decide on a data sharing plan before acceptance, as the process can be lengthy and hold up publication timelines. Please note that, though access restrictions are acceptable now, your entire data will need to be made freely accessible if your manuscript is accepted for publication. This policy applies to all data except where public deposition would breach compliance with the protocol approved by your research ethics board. If you are unable to adhere to our open data policy, please kindly revise your statement to explain your reasoning and we will seek the editor's input on an exemption. Please be assured that, once you have provided your new statement, the assessment of your exemption will not hold up the peer review process.

**Answer #4.** As recommended, we have established a data sharing plan and are currently in the process of depositing our data on the platform [https://recherche.data.gouv.fr/fr](https://recherche.data.gouv.fr/fr?utm_source=chatgpt.com). The entire dataset will be made freely accessible upon acceptance of the manuscript for publication.

**Comment #5.** We note that Figure 1 in your submission contain [map/satellite] images which may be copyrighted. All PLOS content is published under the Creative Commons Attribution License (CC BY 4.0), which means that the manuscript, images, and Supporting Information files will be freely available online, and any third party is permitted to access, download, copy, distribute, and use these materials in any way, even commercially, with proper attribution. For these reasons, we cannot publish previously copyrighted maps or satellite images created using proprietary data, such as Google software (Google Maps, Street View, and Earth). For more information, see our copyright guidelines: <http://journals.plos.org/plosone/s/licenses-and-copyright>.

We require you to either (1) present written permission from the copyright holder to publish these figures specifically under the CC BY 4.0 license, or (2) remove the figures from your submission:

**Answer #5.** Due to the difficulty in obtaining the CC BY 4.0 license, we have decided to remove Figure 1 (map) from the revised manuscript.

**Comment #**6. If the reviewer comments include a recommendation to cite specific previously published works, please review and evaluate these publications to determine whether they are relevant and should be cited. There is no requirement to cite these works unless the editor has indicated otherwise.

**Answer #6.** Thank you for notifying us. At this stage, we have not yet received any comments from the reviewers.

**Additional Editor Comments #1.** Fig. 2, Fig. 4 and Fig. 5 appears to have a low resolution (some labels in these figures are almost unreadable); this would hamper the possibility for Reviewers to perform appropriate reviews.
Please, re-sumbit a manuscript with high-resolution images with well-readable labels.

**Answer #.** We have reproduced the concerned figures in accordance with your comment and resubmitted them. However, following the removal of Figure 1, the numbering has been updated as follows: Fig 1 (previously Fig. 2), Fig 3 (previously Fig. 4), and Fig 4 (previously Fig. 5). In addition, we have uploaded the revised figures to the PACE system, and we now have valid TIFF files.
