# Supplementary material for: Diversity of soil bacterial communities in response to fonio (Digitaria exilis Stapf) genotypes and pedoclimatic conditions in Benin
Source: PLoS One. 2026 Jan 30;21(1):e0330794. doi: 10.1371/journal.pone.0330794 (PMC12857997; doi:10.1371/journal.pone.0330794)
Supplement: Attachment — Submitted filename: Response_to_Reviewers_auresp_2.docx [file pone.0330794.s004.docx]

**Manuscript: Diversity of soil bacterial communities in response to fonio (*Digitaria exilis* Stapf) genotypes and pedoclimatic conditions in Benin**

**Response to reviewers’ comments**

We thank the reviewers for their valuable and helpful comments on the manuscript. We provided here a point-by-point response to the comments.

**Reviewer’s comments**

**Reviewer #2**

**Comment #1.** Provide public accession(s) in a recognized repository like NCBI and add the accession numbers.

**Answer #1**. Thank you for asking this. We have started submitting our data online on data.gouv.fr for public access since we submitted our manuscript. Here is the DOI of the data used in our study: [https://doi.org/10.57745/FQAJBU](https://doi.org/10.57745/FQAJBU).

We also added this link in the methodology part of the manuscript as follows:

**Lines 166:** “The data resulting from this sequencing were available on <https://doi.org/10.57745/FQAJBU>.”

**Comment #**2. The choice to filter at 95% identity is unconventional for 16S ASV workflows. Authors must justify this threshold, clarify its impact on taxonomic resolution, and either reprocess with a stricter threshold or demonstrate that conclusions are unchanged.

**Answer #2**. In our study, we used a 95% identity threshold for data analysis. Although this threshold is slightly lower than the commonly used 97% threshold, it does not affect the overall trend of the results. Firstly, applying the 95% threshold results in only 53 additional ASVs, representing a marginal difference. Secondly, no significant difference is observed between the two thresholds in terms of alpha diversity indices (see table below). Finally, the Bray–Curtis distances represented by the NMDS show no significant variation between the two approaches: the same effects (particularly the site effect) and the same stress value are observed (see the following figure).

**Table. ANOVA between data at a 95% threshold and a 97% threshold.**

| **Alpha diversity index** | **Pvalue** | **Significance level** |
| --- | --- | --- |
| **S.obs** | 0.109 | NS |
| **S.chao1** | 0.0002 | *** |
| **shannon** | 0.861 | NS |
| **invsimpson** | 0.809 | NS |

**Fig. Non-metric multidimensional scaling plot presenting the Bray-Curtis distance of the bacterial community by locations and fonio genotypes at 97% and 95%**

**Comment #3.** why Archaea were removed (primer bias, negligible counts or what else) and provide summary counts showing they are negligible; otherwise include Archaea or report them separately.

**Answer #3.** The Archaea domain identified in this study accounted for only six ASVs, compared to 1,846 ASVs for the Bacteria domain, representing a negligible proportion (0.32%). The low abundance of archaea in metabarcoding datasets has also been reported by Bahram et al. (2018) and can be explained by their incompatibility with commonly used PCR primers (Baker et al. 2003; Gantner et al. 2011). Furthermore, Hartmann et al. (2010) emphasise that the V1 and V2 regions of the 16S rRNA gene offer better resolution for the detection of Archaea taxa than the V4 and V5 regions used in our study.

**References**

Bahram, M., Hildebrand, F., Forslund, S.K., Anderson, J.L., Soudzilovskaia, N.A., Bodegom, P.M., et al. (2018) Structure and function of the global topsoil microbiome. Nature560: 233–237. https://doi.org/10.1038/s41586-018-0386-6.

Baker, G.C., Smith, J.J., and Cowan, D.A. (2003) Review and re-analysis of domain-specific 16S primers. J Microbiol Methods 55: 541–555.

Hartmann, M., Howes, C.G., Abarenkov, K., Mohn, W.W., and Nilsson, R.H. (2010) V-Xtractor: an open-source, high-throughput software tool to identify and extract hypervariable regions of small subunit (16 S/18 S) ribosomal RNA gene sequences. J Microbiol Methods 83: 250–253.

Gantner, S., Andersson, A.F., Alonso-Sáez, L., and Bertilsson, S. (2011) Novel primers for 16S rRNA-based archaeal community analyses in environmental samples. J Microbiol Methods 84: 12–18.

**Summary counts:**

| Domain | Phylum | Order | Class | Familly | Genus | Specie |
| --- | --- | --- | --- | --- | --- | --- |
| Archaea | Crenarchaeota | Nitrososphaeria | Nitrososphaerales | Nitrososphaeraceae | unknown genus | unknown species |
| Archaea | Crenarchaeota | Nitrososphaeria | Nitrososphaerales | Nitrososphaeraceae | Candidatus Nitrocosmicus | Candidatus Nitrocosmicus oleophilus |
| Archaea | Crenarchaeota | Nitrososphaeria | Nitrososphaerales | Nitrososphaeraceae | Candidatus Nitrocosmicus | unknown species |
| Archaea | Crenarchaeota | Nitrososphaeria | Nitrososphaerales | Nitrososphaeraceae | Candidatus Nitrocosmicus | Candidatus Nitrosocosmicus exaquare |
| Archaea | Crenarchaeota | Nitrososphaeria | Nitrososphaerales | Nitrososphaeraceae | Candidatus Nitrososphaera | unknown species |
| Archaea | Crenarchaeota | Nitrososphaeria | Nitrososphaerales | Nitrososphaeraceae | Candidatus Nitrososphaera | unidentified archaeon SCA11 |

**Comment #4.** Explicitly state the total number of sequenced samples and the sampling unit,

**Answer #4.** In total, 45 rhizosphere soil samples were analyzed by sequencing. The samples represented five fonio genotypes, cultivated in three experimental sites replicated according to three blocks per site. Each sampling unit consisted of a composite sample obtained by pooling rhizospheric soils from three plants of a given genotype. No technical replicates were considered for sequencing.

**Lines 140 – 143:** “At each site, rhizosphere soil was collected from three plants per genotype and pooled to form one composite sample, representing our sampling unit. Three replicated composite samples were collected per site, and 45 samples collected in total (3 sites * 3 replicates * 5 genotypes).”

**Comment #5.** Standardize taxonomic names (spellings such as Weeksellaceae, Actinobacteriota/Actinobacteria); ensure consistent taxonomy source.

**Answer #5.** Thank you for noticing this. For our study, we used the SILVA 138.1 Pintail 100 database. We corrected the error in the spellings used in the manuscript:

“Actinobacteriodata in Actinobacteriota”

“Weekselleceae in Weeksellaceae”

**Comment #6**. Confirm and supply final high-resolution figure files (300–600 dpi.

**Answer #6.** Our graphic files were exported from R software and then uploaded to the Preflight Analysis and Conversion Engine (PACE) digital diagnostic tool (<https://pacev2.apexcovantage.com/>) to ensure that our figures met PLOS requirements. After increasing the resolution of the figures, we re-evaluated them using PACE to confirm compliance with journal standards:

- Fig1. 450 dpi
- Fig2. 450 dpi
- Fig3. 450 dpi
- Fig4. 450 dpi
- Fig5. 330 dpi
- Fig6. 330 dpi

**Comment #7**. Clarify enzyme-assay details (units, dry-weight basis, number of technical replicates).

**Answer #7.** We applied methods defined by Clivot et al. (2020)**.** We added this in the manuscript at “**Determination of soil parameters**” section**:**

**Lines 183 - 193: “**4 g of fresh soil was mixed with 50 ml of 0.1 M Tris-HCl buffer pH 8.0, and diluted at a ratio of 1:100. In a microplate, 100 µL of 200 µM substrate (defined according to the enzyme to be measured, Table 3) was then added to 100 µL of the sol solution; four replicates were performed per sample. A standard curve is also produced using 4-methylumbelliferone (MUF) or 7-amino-4-methylcoumarin (AMC) (Table 3) at concentrations of 0 µM, 2.5 µM, 10 µM, and 25 µM; 100 µL of each concentration was added to 100 µL of the soil solution. The mixture was left to incubate for varying lengths of time depending on the enzyme (Table 3). After incubation at 37°C, enzyme activities were measured using fluorimetry by taking a reading at an excitation wavelength of 360 nm and an emission wavelength of 460 nm. Values were then expressed in micromoles per hour per gram of dry equivalent soil (µmol/h/g).”

**Table 3. The enzymes determined from fonio rhizosphere soil samples and the corresponding substrates. 4-methylumbelliferone (MUF), 7-amino-4-methylcoumarin (AMC).**

| **Enzymes measured** | **Substrates** | **Incubation time** | **Standard used** |
| --- | --- | --- | --- |
| **Arylsulfatase (ARS)** | 4-Methylumbelliferyl-sulfate | 6 hours | MUF |
| **β -glucosidase (BGLU)** | 4-Methylumbelliferyl- β -D-glucopyranoside | 2 hours | MUF |
| **Leucine aminopeptidase (LAP)** | L-Leucine-7-amido-4-methylcoumarin | 3 hours | AMC |
| **β-N-acetylglucosaminidase (NAG)** | 4-Methylumbelliferyl-N-acetyl- β -D-glucopyranoside | 6 hours | MUF |
| **Phosphatase (P and Palc)** | 4-Methylumbelliferyl-phosphate | 2 hours | MUF |
| **Xylosidase (xylo)** | 4-Methylumbelliferyl-B-D-xylopynanoside | 6 hours | MUF |

**Comment #8**. Rephrase sentences to clearly distinguish alpha (no significant effect) versus beta (significant location effect) diversity results.

**Answer #8.** The results about alpha and beta diversity were reformulated as following:

**Lines 293 - 297:** “The alpha-diversity indices were calculated and presented in Table 5. The results revealed that there are no significant differences among locations and genotypes for all alpha-diversity indices assessed (Observed ASV, Chao1 estimator, inverse Simpson index and Shannon index). Observed ASV ranged in average from 754.6 (Ina) to 788.67 (Gogounou) and from 760.30 (MAL14) to 791.44 (MAL05) for location and genotypes, respectively.”

**Lines 308 - 313:** “According to a PERMANOVA applied on the beta-diversity (Table 6), the location explained 22% of the variation in bacterial communities, the genotype explained 7.8% and the interaction of location and genotype explained 15.3%. However, neither genotype nor the interaction had a significant influence on the diversity and community structure of fonio rhizosphere bacteria (P > 0.05). Only the location recorded a significant effect on the bacterial community structure (P < 0.001).”

**Comment #9**. Consider functional inference (PICRUSt2/Tax4Fun) or correlation analyses between microbiome features and genotype performance (yield/height) to strengthen biological interpretation.
**Answer #9.** Thank you for this insightful comment. We performed a functional inference with Tax4Fun in R software as suggested.

**Bioinformatic and statistical analysis section:**

**Lines 227 – 230:** “Furthermore, the potential functions of the bacterial communities were inferred from the 16S rRNA gene sequences using the Tax4Fun2 [51], which predicts functional profiles by mapping taxa to the KEGG (Kyoto Encyclopedia of Genes and Genomes) Orthology database for prokaryotes.”

**Results section:** Function prediction of bacterial community in fonio rhizosphere

**Lines 392 - 430:** “A variety of functional traits of the fonio rhizosphere bacterial community were identified using Tax4Fun2 analysis. In total, 8,283 KEGG orthologs (KO) were detected across all samples and classified into six KEGG level 3 functional categories: metabolism (52.80%), environmental information processing (23.13%), cellular processes (13.08%), genetic information processing (6.79%), human diseases (3.25%), and organismal systems (0.95%). Among these, metabolism represented the most abundant functional module across the three study locations. Location had no significant effect on metabolism, environmental information processing, and human diseases (P > 0.05) (Table S1). In contrast, significant location effects were observed for cellular processes (P = 0.03), genetic information processing (P < 0.01), and organismal systems (P < 0.001) (Table S1). Functional traits related to genetic information processing and organismal systems were particularly more abundant at the Boukoumbe site (Fig 5).

At the KEGG level 2, 46 KO groups were identified. Functions associated with global and overview maps were the most abundant (23.54%), followed by membrane transport (12.49%), signal transduction (10.64%), cellular community of prokaryotes (9.35%), carbohydrate metabolism (6.51%), and amino acid metabolism (5.15%) (Fig 6). Additional gene functions related to energy metabolism, lipid metabolism, xenobiotic degradation, nucleotide metabolism, terpenoid and polyketide metabolism, cofactor and vitamin metabolism, translation, replication and repair, cell motility, antimicrobial drug resistance, protein folding, sorting and degradation, cell growth and death, and glycan biosynthesis and metabolism were also detected. Significant differences among locations were observed for glycan biosynthesis and metabolism, cell growth and death, folding, sorting and degradation, metabolism of terpenoids and polyketides, xenobiotic biodegradation and metabolism, replication and repair, energy metabolism, carbohydrate metabolism, and cellular community of prokaryotes (Table S2). Specifically, functions related to glycan biosynthesis and metabolism, folding, sorting and degradation, replication and repair, energy metabolism, carbohydrate metabolism, and cellular community of prokaryotes were significantly more abundant at the Boukoumbe site, whereas metabolism of terpenoids and polyketides, xenobiotic biodegradation and metabolism, and energy metabolism were more enriched at the Ina site.

Fig 5. Functional traits related to level 3 KEGG pathways in the fonio rhizosphere bacterial community based on Tax4Fun2 analysis. Values designated by the same letters are not significantly different (Tukey HSD test; P ≤ 0.05).

Fig 6. Functional traits related to level 2 KEGG pathways in the fonio rhizosphere bacterial community based on Tax4Fun2 analysis. Functions related to metabolism (a), functions related to environmental information processing (b) and functions related to cellular processes (c).”

**Discussion section:** Soil bacterial community structure, composition (abundance and diversity) and functions
**Lines 498 – 522:** “These communities associated with the fonio rhizosphere may be involved in processes relevant to plant growth and soil functioning. Based on Tax4Fun2 analysis, six major functional modules were detected in the samples, with metabolism, environmental information processing, and cellular processes being predominant across all locations. Similar gene functions have also been reported by Adigoun et al. (2025) in Synsepalum dulcificum roots and by Dong et al. (2020) in cave environments. Moreover, Yang and Song (2019) reported the predominance of metabolism-related functions during the degradation of solid wastes, where microbial communities are typically enriched in genes involved in nutrient transformation, carbon cycling, and energy metabolism. Such a high metabolic potential indicates that the fonio rhizosphere harbors an active bacterial community capable of supporting plant nutrition and soil fertility through organic matter degradation and nutrient mineralization. Although location had no significant influence on the abundance of metabolism, environmental information processing, and human disease-related functions, the significant effects observed on cellular processes, genetic information processing, and organismal systems suggest that abiotic factors, such as soil pH, play a role in shaping specific microbial functional traits [77]. The higher abundance of genes related to genetic information processing and organismal systems at the Boukoumbe site may reflect enhanced microbial activity, consistent with the higher enzymatic activities recorded at this location. At KEGG level 2, the dominance of functions related to global and overview maps, membrane transport, and signal transduction suggests an active exchange of metabolites and signalling molecules within the rhizosphere. These processes are crucial for maintaining both plant-microorganism and microorganism-microorganism interactions, particularly in nutrient-limited soils [78]. Furthermore, the abundance of genes related to carbohydrate and amino acid metabolism in the rhizosphere supports the role of the bacterial community in decomposing root exudates and soil organic matter, thus contributing to carbon and nitrogen cycling [79].

**Reviewer #3**

**Overall Comments**
The manuscript would benefit from (i) consistent use of 16S terminology, (ii) a brief justification of sequencing depth, and (iii) clearer reporting for enzyme activities (comparators, units, and tests). In places where biological interpretation relies on broad taxonomic ranks, adding genus-/strain-level anchors (or clearly flagging speculation) will strengthen the narrative.

**Line-Specific Comments**

**Comment # Line 103:**
Please use consistent terminology: 16S rRNA gene (i.e., 16S rDNA). Avoid forms such as “16S rDNA genes”.

**Answer#.** Thank you for this valuable comment, which helped improve the rigor and overall quality of the paper. The terminology has been corrected accordingly in the revised version.

**Comment # Line 193:**
How was the 11,057 reads/sample threshold determined? Please justify the cutoff (e.g., rarefaction curves, median depth, sensitivity to alternative thresholds) and list any samples excluded due to insufficient depth.

**Answer#.** The number of reads per sample was rarefied to 11,057 representative sequences using the following functions in R software:

rowSums()

min(rowSums()) :11,057 in our study

rrarefy()

No sample was excluded, as rarefaction was performed according to the minimum number of reads observed across all samples.

**Comment # Lines 391–393:**
“Higher/lower activity” is ambiguous. Higher/lower relative to which condition/site/timepoint? Specify assay conditions and units (substrate, temperature/pH, normalization basis), n and variability, effect sizes, and the statistical test/model (with multiple-testing correction, e.g., FDR). Avoid comparing magnitudes across different enzymes unless activities are placed on a common scale.

**Answer#.** In the “**Determination of soil parameters**” section, we have now included a detailed description of the methodology used to determine soil enzyme activities, explaining the conditions (**lines** **180 - 197**). In addition, the corresponding results have been rephrased to better reflect your suggestions:

**Lines 281 – 285:** “As shown in Table 5, soil enzyme activities varied significantly among locations (P < 0.001), whereas no significant effect of fonio genotype was detected (P > 0.05). Both alkaline and acid phosphatase activities were significantly higher at Ina and Boukoumbe compared to Gogounou. Similarly, ARS, BGLU, LAP, NAG, and xylosidase activities exhibited significantly greater values at Boukoumbe than at the other sites.”

**Lines 408:** “We recorded a higher enzyme activity in the Boukoumbe site than in the other sites.”

**Comment # Line 405:**
Do you mean high activity at all sites? Provide summary statistics (central tendency ± variability, n per site) and results of formal tests. If only some sites show elevated activity, qualify the statement accordingly.

**Answer#.** Yes, we recorded high values for the phosphatase activities in our study.

**Correction on lines 419 - 421**: “Phosphatases (P and Palc) contribute to the mineralization of organic phosphorus and are more prevalent at all locations here [26,59].**”**

**Comment # Line 415:**
Inferences at phylum/class (even family) level are too coarse to link relative abundance to drought resistance or other beneficial phenotypes. Either provide genus- or strain-level evidence (e.g., taxa with documented PGP traits, with citations) or clearly label the interpretation as speculative. Strengthen with effect sizes and FDR-adjusted p-values; the current text is qualitative.

**Answer#.** Function inference was finally performed and added to the manuscript for this purpose. See lines **392 – 430** and **lines** **498 – 522** for the results and discussion.

**Comment # Line 420:**
“Composition of significant phyla” is unclear. If you mean phyla whose relative abundances differ significantly among groups, please state it explicitly and specify the taxonomic rank, statistical test, significance threshold, and multiple-testing correction.

**Answer#.** Thank you for this remark. In this sentence, we were referring to the most abundant phyla observed in the rhizosphere of other millet species.

**Correction on lines 436 – 437:** “The most abundant phyla vary significantly among different millet species, as pointed out by Tian et al. (2022) for proso millet, foxtail millet and sorghum.”

**# Changes in references list**

In light of certain comments made by reviewers, some parts of the discussion have been removed, involving the removal of certain references and the addition of new ones. The references have therefore been updated in the manuscript.
