# Supplementary material for: Diversity of soil bacterial communities in response to fonio (Digitaria exilis Stapf) genotypes and pedoclimatic conditions in Benin
Source: PLoS One. 2026 Jan 30;21(1):e0330794. doi: 10.1371/journal.pone.0330794 (PMC12857997; doi:10.1371/journal.pone.0330794)
Supplement: Attachment — Submitted filename: Response_to_Reviewers_auresp_3.docx [file pone.0330794.s005.docx]

**Manuscript: Diversity of soil bacterial communities in response to fonio (*Digitaria exilis* Stapf) genotypes and pedoclimatic conditions in Benin**

**Response to reviewers’ comments**

We thank the reviewers for their valuable and helpful comments on the manuscript. We provided here a point-by-point response to the comments.

**Reviewer’s comments**

**Reviewer #2**

**Comment #1.** The introduction excellently establishes the context of plant-microbe interactions and the importance of location vs. genotype. However, the specific rationale for hypothesizing a genotype effect in fonio is slightly underdeveloped. Briefly elaborate on why these specific agronomic traits (e.g., maturity cycle, plant height) might be expected to influence rhizosphere communities, perhaps via differences in root exudation patterns or growth dynamics, to better justify the genotype-related hypotheses.

**Answer #1.**  We have added a study that supports this hypothesis.

**Lines 80 – 84: “**For example, wheat genotypes that differ in plant height show distinct bacterial communities in their rhizosphere (Kavamura et al., 2020). Furthermore, due to their genetic variability, genotypes may exhibit different functional characteristics that could potentially explain the release of specific root metabolite profiles, thereby creating distinct microhabitats leading to differentiated microbial assemblages.”

**Comment #2.** Three replicated composite samples were collected per site..." This description is ambiguous. Does this mean there were 9 plants per genotype per location (3 plants x 3 composite samples)? Or were the three plants pooled to make one composite, and this was repeated three times (i.e., 9 plants total per genotype per location)?

**Answer #2.** Three plants were combined to form one composite.

**Lines 143 – 145:** “At each site, rhizosphere soil was collected from three plants per genotype per replication, pooled to form one composite sample, representing our sampling unit. Then, three composite samples were collected per site, and 45 samples collected in total”

**Comment #3.** In the Results (Fig 3b), it is stated that "Proteobacteria and Bacteroidota were more abundant at Gogounou (52.5%) and at Boukoumbe (34.7%) than at other locations, respectively." However, in the Discussion, the high abundance of Firmicutes at the most acidic site (Ina) is highlighted. The text would benefit from explicitly stating the actual measured abundance of Firmicutes at Ina compared to other sites to strengthen this observation.

**Answer #3.** Thank you for noticing that.

**Lines 330 - 332:** “Regarding the third most abundant bacterial community, Firmicutes, their relative abundance was significantly higher at Ina (9.3%) than at other pedoclimatic conditions (4.9% at Boukoumbe and 4.3% at Gogounou)”.

**Comment #5.** The conclusion is strong but does not explicitly return to the four initial hypotheses to state which were supported and which were not.

**Answer #5.** The conclusion has been corrected based on your comment.

**Lines 584 - 594:** “Our study provides a comprehensive overview of the rhizosphere soil bacterial community associated with fonio across agroecological zones in northern Benin. We hypothesized that the chemical and microbial properties of fonio rhizosphere soils depend primarily on pedoclimatic conditions; this hypothesis is supported. We also expected that bacterial community structure would be shaped by both pedoclimatic conditions and plant genotype; this assumption is only partially validated. In fact, bacterial community structure in the fonio rhizosphere was influenced particularly by soil parameters, thus supporting our third hypothesis. Finally, we proposed that these communities would exhibit diverse functions related to soil health and fertility, and this hypothesis is confirmed. Together, these outcomes clarify the role of the rhizosphere microbiote and offer prospects for future research aimed at enhancing crop productivity.”

**Comment #6.** To explain the lack of a genotype effect, the authors hypothesize that "the fonio genotypes studied here exhibit genetic similarity." This is a reasonable speculation, but no data on the genetic relatedness of these genotypes is provided. They originate from different countries (Benin, Mali, Niger), so they may not be genetically similar.

**Answer #6.** We have revised this part based on your comment.

**Lines 549 - 557:** “The genotypes used in this study may exhibit similar root architecture and root exudate profiles, thereby reducing their influence on microbial community structure. This is consistent with the hot water extractable soluble carbon and nitrogen contents measured, used as indicators of rhizodeposition, which did not vary significantly among fonio genotypes. Several studies have also shown that soil microbial communities structure varies according to the plant developmental stage, with root exudates exerting a particularly strong influence during early growth phases [83]. In our study, sampling was conducted at a late developmental stage, which likely reduced the strength of exudate-driven selection effects.
